# The update and optimization of an eDNA assay to detect the invasive rusty crayfish (*Faxonius rusticus*)

**DOI:** 10.1371/journal.pone.0259084

**Published:** 2021-10-29

**Authors:** Stephanie S. Coster, Megan N. Dillon, William Moore, George T. Merovich

**Affiliations:** 1 Department of Biology, Randolph-Macon College, Ashland, Virginia, United States of America; 2 Department of Environmental Science, Fisheries and Aquatic Sciences Program, Juniata College, Huntingdon, Pennsylvania, United States of America; University of Hyogo, JAPAN

## Abstract

Environmental DNA (eDNA) is nuclear or mitochondrial DNA shed into the environment, and amplifying this DNA can serve as a reliable, noninvasive way to monitor aquatic systems for the presence of an invasive species. Assays based on the collection of eDNA are becoming increasingly popular, and, when optimized, can aid in effectively and efficiently tracking invasion fronts. We set out to update an eDNA assay to detect the invasive rusty crayfish, *Faxonius rusticus*. We tested for species specificity compared to other stream crayfish and field tested the assay at sites with known presence (N = 3) and absence (N = 4) in the Juniata River watershed in central Pennsylvania, USA. To maximize sensitivity, we field tested different storage buffers (Longmire’s buffer and ethanol), DNA extraction methods (Qiagen’s DNEasy and PowerWater kits), and quantitative polymerase chain reaction (qPCR) chemistries (TaqMan and SYBR green). Our assay confirmed the presence data and performed optimally when filter samples were stored in Longmire’s buffer, DNA was extracted with DNeasy Blood and Tissue Kit, and TaqMan qPCR chemistry was utilized. With proper sample processing, our assay allows for accurate, noninvasive detection of *F*. *rusticus* in streams.

## Introduction

The invasion of non-native species is widespread and can have far reaching negative consequences for ecosystems world-wide. The detrimental impacts range from the disruption of food webs and the reorganization of biological communities [1, 2] to economic losses stemming from shifts in resource use in agricultural, recreational, fishery, or forestry sectors [3, 4]. Freshwater aquatic ecosystems are especially susceptible to the introduction of invasive species through ballast water transfer, stocking by natural resource managers, horticulture contamination, or emptying bait buckets after recreational fishing [2]. Over the last century, freshwater ecosystems across North America have experienced a number of invasions from different taxa that have disrupted ecosystem balance. A few notable examples include the zebra mussel (*Dreissena polymorpha)*, the sea lamprey (*Petromyzon marinus*), and the rusty crayfish *Faxonius rusticus* [2]. The destabilizing effects of these invasions vary. For example, the zebra mussel filters large amounts of phytoplankton which results in the pelagic food web languishing and the littoral food web thriving [5]. The emigration of the parasitic sea lamprey into the Great Lakes in the early 20^th^ century caused a sharp decline in predatory species such as the lake trout (*Salvelinus namaycush*) and a dramatic shift in community composition [6–8]. Finally, the introduction of the omnivorous and aggressive rusty crayfish (*Faxonius rusticus*) [9] into freshwater systems beyond their native range leads to a reduction in macrophyte biomass [10, 11] and a shift the composition of other benthic macro-invertebrates [12, 13]. In addition, rusty crayfish are a threat to native species as they have higher growth rates and outcompete natives [10, 14] and have been found to hybridize with native species, which can lead to genetic swamping [15].

The early detection of new invasions and subsequent surveillance of these and older invasions is key to implementing management strategies to limit the harmful impacts of these species. A promising tool that has gained momentum in the last decade is the use of environmental DNA (eDNA) to investigate species presence [16–18], estimate biomass/abundance [19–23], or to identify community composition [24] of freshwater species. eDNA methods capture and amplify trace DNA in the environment generated from cells of microorganisms or shed from more complex organisms in mucous, excrement, tissue, or during decay [25]. The DNA of interest can be captured from soil or sediment [26], water [17], swabs [27], or feces [28]. Quantitative pcr (qPCR) and digital droplet PCR are primarily used to amplify eDNA products in species-specific assays, while high-throughput sequencing and metagenomics is used in understanding community composition [25].

The steps of a single-species eDNA monitoring protocol can be broken down into several stages: sample collection, sample transport/storage, DNA extraction, and qPCR amplification/analysis [29]. At each stage there are multiple options that need to be considered in order to optimize detection of the target species at the sampling location [30]. In the field, DNA fragments from water samples are captured by precipitation [16, 18] or filtration [17, 31]. Cellulose, glass fiber, polycarbonate, nylon, and polyethersulfone filters are all effective for eDNA capture [30, 32]. Additionally, the pore size of the filter is an important consideration as there is a trade-off between clogging with smaller pore sizes and less exogenous DNA capture at larger pore sizes [29]. Options for sample storage include freezing or desiccating filters or placing them in a storage buffer [30, 33]. While ethanol has long been used for DNA storage and has been successful for eDNA filter preservation [17], Longmire’s buffer has been proposed as an alternative and has been successful, and sometimes better, for eDNA analyses [33, 34].

After water samples have been filtered and brought back to the lab, the next step is to extract DNA from the filters [29]. Options here include phase separation/precipitation-based protocols or using one of a number of different extraction kits that may have varied levels of success [29]. Phenol-chloroform and CTAB methods have both been successful for extraction in preparation for eDNA assays [33, 35]. In addition, Qiagen DNeasy Blood and Tissue (hereafter DNeasy) and Qiagen DNeasy PowerWater (hereafter PowerWater) extraction kits allow for a more standardized, albeit more expensive, extraction methodology that have also proved useful in eDNA studies [35, 36].

In addition to primer development and sample handling, qPCR chemistry can impact the results of eDNA assays [37]. The two most common options are qPCR assays using SYBR green or TaqMan chemistry. SYBR green is an intercalating dye that fluoresces in the presences of any double-stranded DNA including non-target organisms. It is cheaper but can result in false positives if there is non-specific DNA that amplifies. A TaqMan assay requires fluorescent primers and an internal probe that results in greater specificity for the target organism and the ability to add an internal positive control (IPC) to assess sample inhibition but is more costly. A TaqMan assay is highly recommended for eDNA detection because of its low probability of cross amplification [29]. Invariably, methods and techniques improve as the field develops, and it is important to update protocols that were published in the early days of research in this field.

In 2016, Dougherty et al. [38] were the first to publish a field validated eDNA assay to detect the invasive rusty crayfish in lakes of the Midwest, USA. The rusty crayfish, native to the Ohio River Valley, is invasive in areas outside of its range [14, 39] and has spread across the north and northeast of the United States, including the Susquehanna River basin at least since the 1960s and more recently in the Juniata River sub-basin in central Pennsylvania, USA [11, 40, 41]. The invasion in the Juniata River watershed is of particular interest due to the economic importance of recreational fishing in Raystown Lake and the Little Juniata River, a renowned cold-water trout fishery. Invasion of *F*. *rusticus* in these aquatic systems would not only harm the native community, but could impact the local economy in Blair County, Pennsylvania [42].

Our goal was to update the eDNA assay of Dougherty et al. [38] using contemporary eDNA practices and to test it in streams so that it can be used to track the invasion front of rusty crayfish in the Juniata River watershed. Our specific objectives for this study were to (1) develop a TaqMan qPCR primer pair and probe specific to *F*. *rusticus*; (2) field test the qPCR assay to ensure sensitivity to detect *F*. *rusticus* at sites with known presence; and (3) test different eDNA storage techniques, extraction methods, and qPCR chemistry to optimize the eDNA assay.

## Materials and methods

### Developing qPCR assay & testing sensitivity

To develop the eDNA assay, we modified a primer pair from Dougherty et al. [38] targeting the cytochrome oxidase I (COI) gene region of *F*. *rusticus* by clipping the primer to avoid the instability of a guanine tetrad, hereafter referred to as FaRu. The FaRu primers amplified 112bp of the COI gene and the sequences of primers were FaRu-F 5’-GGGCGTCAGTAGATTTAGGTATT-3’ and FaRu-R 5’-GTCATTCCCGTAGCTCGTATATT-3’. We utilized the Sequence Manipulation Suite PCR Products tool [43] to complete in silico testing of this primer, against 14 stream crayfish species that inhabit Pennsylvania (S1 Table). To further test the primers for specificity to samples in the Juniata watershed in central Pennsylvania, USA we obtained tissue from three different individuals from three different crayfish species, *F*. *rusticus*, Appalachian brook crayfish *(F*. *obscurus)*, and Eastern crayfish *(Cambarus bartonii*). Tissue samples were preserved in 95% ethanol prior to extractions. We extracted genomic DNA using a DNeasy Blood and Tissue extraction kit (QIAGEN Inc.) following the protocol. We ran a traditional PCR in a 20 uL reaction with 1X GoTaq Flexi buffer, 0.16 mg/mL BSA, 0.2 mM dNTPs, 3 mM MgCl_2_, 0.2 uL of Taq polymerase, 20 ng of template DNA and water. We used a touchdown PCR program in a Bio-Rad T100 thermocycler, starting with 5 minutes at 95°C, and then 13 cycles of 95°C for 30s, 65°C for 30s, and 72°C for 90s, with each cycle having a lower annealing temperature by 1 degree; after arriving at an annealing temperature of 53°C, there were 24 cycles of 95°C for 30s, 53°C for 30s, and 72°C for 90s, followed by 72°C for 10 minutes and an infinite hold at 4°C. The PCR products were run on 2% agarose gel electrophoresis for visualization.

Using Integrated DNA Technology’s PrimerQuest tool we identified a TaqMan probe sequence based on documented *F*. *rusticus* (GenBank Accession no. AY701249). The FaRu probe used was 5’-ACTGAGCCAAGAATAGAAGAAACCC-3’. To test the TaqMan qPCR assay we ran all tissue samples in triplicate; in a 20 μL reaction we used 10 uL of Integrated DNA Technology’s PrimeTime Master Mix buffer, 2 uL of FaRu PrimeTime Probe Assay and the IPC primer, 2 uL of the IPC primer/probe assay, 0.4 uL of IPC template DNA, 4 uL of crayfish template DNA (diluted to mimic low concentration in water samples) and water. We included an internal positive control (IPC; TaqMan Exogenous Internal Positive Control, Fisher Scientific) in the assay to evaluate sample inhibition and included a negative control. We used a StepOnePlus thermocycler (Applied Biosystems) with 3 min at 95°C followed by 40 cycles of 95°C for 15 sec and 60°C for 1 min. We averaged cycle-threshold (C_T_) values of all replicates for each species to confirm specificity.

There is a 4 bp difference between *F*. *rusticus* and *F*. *obscurus* in the COI sequences in our FaRu primers. To investigate whether we could distinguish *F*. *rusticus* and *F*. *obscurus* tissue from their melt curves, we also ran a qPCR reaction with SYBR green and conducted a one-way ANOVA in Excel (*α* = 0.05) to compare mean T_M_. Each reaction contained 10 μL SyGreen Mix Hi-Rox (PCR Biosystems), 0.3 μM forward primer, 0.3 μM reverse primer, 4 μL of template DNA and water to bring the final volume to 20 μL. We utilized the following qPCR program: 30 sec at 60°C, 10 min. at 95°C, 40 cycles of 95°C for 15 sec and 62°C for 1 min, followed by 30 sec at 60°C followed by a melt-curve analysis.

### Field testing and optimization

To validate the eDNA assay we focused our sampling efforts in the upper Juniata River watershed near Raystown Lake, Pennsylvania where *F*. *rusticus* have been introduced. We first identified sites based on prior information about *F*. *rusticus* presence that used dip net capture methods. We identified sites as known presence or absence or unknown (if the sites had not been previously surveyed). All sampling of crayfish was authorized by the Pennsylvania Fish and Boat Commission under scientific collecting permit number 2020-01-0086. All sampling locations were accessed via public rights-of-way, fishing access points, or municipal parks. In total, 8 sites were sampled: 3 sites of known presence, 4 sites of putative absence, and one site with unknown presence (Table 1). One of the positive sites, Shaver’s Creek, is the only site with presence of both *F*. *rusticus* and *F*. *obscurus*.

**Table 1:** Sampling sites for eDNA assay in the Juniata River watershed, Pennsylvania.

Site	Presence^a^	Lat	Long
Frankstown Branch Mouth (FM)	A	40.536736	-78.077763
Upper Juniata River (UJ)	P	40.538297	-78.030638
Upper Little Juniata River (LJU)	A	40.587196	-78.090486
Shaver’s Creek (SC)	B	40.5841	-78.0563
Little Juniata River at mouth (MLJ)	A	40.561822	-78.068121
Trough Creek (TC)	A	40.31019	-78.127942
Aughwick Creek (AC)	U	40.356598	-77.841185
Lower Juniata River (MJ)	P	40.48527	-78.018414

^a^ A is putative absence, P is presence, and U is unknown.

For water sample collection, we utilized disposable Whatman cellulose nitrate filters (pore size of 0.45 μm) in filter funnels attached to tubing connected to a peristaltic pump. We placed the filter funnel into the stream in areas with riffles due to benthic substrate, including rocks that provide shelter for crayfish, and ran stream water through until clogged or one liter had passed through (measured as output into a collection container). At each site, we ran this protocol with four filters and then used tweezers to remove the filter and place them in microcentrifuge tubes containing preservation buffer. To prevent cross-contamination, we rinsed the tweezers with 10% bleach and gloves were changed for each sample.

To find the most effective preservation buffer, two eDNA samples collected from each location were stored using Longmire’s Buffer and two in 95% ethanol. For each preservation buffer, we utilized both DNeasy PowerWater Kit (QIAGEN Inc.) and DNeasy Blood and Tissue Kit to compare their effectiveness in extracting the eDNA from the water samples. We followed the manufacturer’s instructions for the PowerWater kit. We added an additional homogenization step with PowerBead tubes (QIAGEN Inc.) to the DNeasy Blood and Tissue procedure as follows. We added filters to the PowerBead tubes with 900 μL of ATL buffer, vortexed at max speed for 5 min., incubated at 56°C for 30 min., and then this was repeated. We incubated for 2 hours after addition of 100 μL of Proteinase K. We centrifuged samples at 10,000 g for 1 min. and transferred the supernatant to a new tube with 650 μL of AL buffer and 650 μL of 95% ethanol prior to following the Qiagen guidelines for washing. A final elution volume of 100 μL (50 μL twice) was utilized for both protocols.

We performed an LOD and LOQ analysis with a minimum of 10 replicates of 10 gBlock (IDT) standards (520 bp from GenBank Accession no. AY701249) using a 1:10 dilution factor (range 1 – 1E^-9^ ng/μL gene fragments, or 10E^9^–10 copies/reaction). We used the curve-fitting model approach of Klymus et al. [44] to determine the LOD and LOQ using their R script in RStudio [45]. Similar to Carim et al. [46], we utilized the putative negative sites as field controls. For each combination of preservation and extraction techniques, we averaged C_T_ values across replicates, and compared amplification success. As recommended by MIQE guidelines [47], we utilized a C_T_ cutoff value to distinguish a positive signal from background [48–50] based on the LOD for the Taq Man assay (S1–S3 Figs). We could not determine an LOD for the SYBR assay because our lowest standard had >95% replicates amplify. We therefore chose a C_T_ cutoff value of 32 based on where the C_T_ value plateaued across our lowest standards (S4 Fig). The criteria for determining the number of positive technical replicates needed to infer species presence varies across studies [29]. We therefore determined *F*. *rusticus* was present when 3 out of 6 replicates in the TaqMan assay had a C_T_ value of < 34 and the Sybr assay had a C_T_ value of < 32.

We performed qPCR on filtered water samples with the aforementioned TaqMan probe assay using the same reaction set up with 4 uL of extracted eDNA template with six replicates per sample. We added 40 μL ROX dye to 1 mL PrimeTime master mix stock, which served as a passive standard. We ran each plate following the previously noted protocol, in addition to five gBlock (IDT) standards using a 1:10 dilution factor (range 1E^-1^ – 1E^-5^ ng/μL gene fragments or 9E^8^ – 9E^4^ copies/reaction), a PCR negative control, an extraction negative control, and an IPC blocked control. To compare the sensitivity of qPCR chemistry we also tested a SYBR green assay using 10 μL of SyGreen Mix Hi-Rox (PCR Biosystems), 0.3 μM FaRu forward and reverse primers and water in a 20 μL reaction with 4 μL of extracted eDNA template using the aforementioned qPCR program. We did not include an IPC because it would have interfered with the signal. We ran a melt curve on each plate to assess the melt profile of each eDNA filter sample.

## Results

### Developing qPCR assay & testing sensitivity

In the traditional PCR the FaRu primer amplified only the *F*. *rusticus* tissue samples and did not amplify the other two species. Likewise, the TaqMan qPCR primer and probe amplified the known *F*. *rusticus* tissue samples and did not amplify the other two species. The negative controls run with the tissue samples had no amplification lower than the C_T_ threshold, and the IPC showed consistent amplification through all samples and replicates, suggesting that there was no inhibition. The SYBR green analysis with tissue samples had amplification of all three samples of *F*. *rusticus* and *F*. *obscurus*, but only 2/3 samples of *C*. *bartonii*. The negative controls had no amplification lower than the C_T_ threshold. There was no clear distinction in the T_M_ between species (by ANOVA, F(2, 24) = 1.88, p = 0.17).

### Field testing and optimization

**Fig 1:**
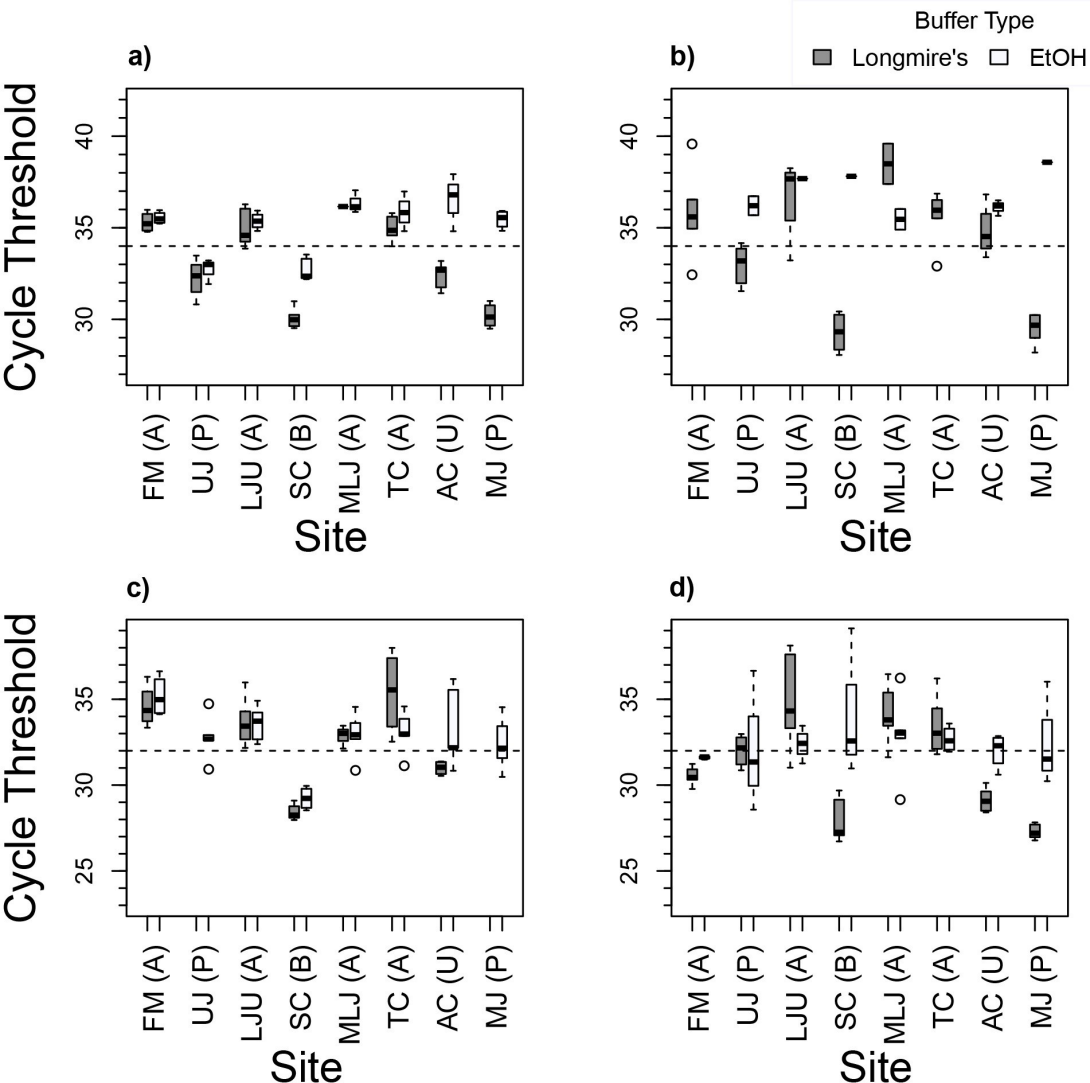
Average cycle threshold values following qPCR amplification with FaRu primers. (A) Sample DNA was extracted with DNeasy and TaqMan chemistry was used, (B) PowerWater TaqMan, (C) DNeasy SYBR, (D) PowerWater SYBR. Cutoff values set at 34 for TaqMan chemistry (A) and (B) and 32 for SYBR (C) and (D). Site codes as in Table 1. Presence is noted as A for putative absence, P for presence, B for both *F*. *rusticus* and *F*. *obscurus*, and U for unknown.

Utilizing the DNeasy Blood and Tissue extraction kit and Longmire’s buffer, putative presence information was confirmed with the FaRu TaqMan assay. Samples from the three putative positive locations (upper Juniata River, Shaver’s Creek, and lower Juniata River) generated average C_T_ values below the 34-cycle threshold (Fig 1A, Table 2). The sample from Aughwick Creek, where presence was unknown, resulted in an average C_T_ of 32.4, and this amplification below the cutoff threshold suggests *F*. *rusticus* was present at that site (subsequent field visits verified its presence). This extraction kit and preservation buffer combination confirmed sites with putative absence, with the samples from Frankstown Branch mouth, Little Juniata River upper, Little Juniata River mouth, and Trough Creek showing amplification after the C_T_ 34 threshold.

**Table 2:** Qiagen’s DNeasy Blood and Tissue extraction kit with a TaqMan qPCR assay.

DNeasy, TaqMan							
		Longmire’s Buffer			95% Ethanol		
Site	Presence^a^	Conc. (nmol)	Avg. C_T_	C_T_ = 34	Conc. (nmol)	Avg. C_T_	C_T_ = 34
Frankstown Branch Mouth (FM)	A	1.5 E-7	35.2	0/6	1.5 E-7	35.5	0/6
Upper Juniata River (UJ)	P	9.7 E-7	32.3	**6/6:**	1.4 E-6	32.7	**3/6:**
Upper Little Juniata River (LJU)	A	2.2 E-7	34.9	1/6:	1.5 E-7	35.4	0/6
Shaver’s Creek (SC)	B	3.5 E-6	30.1	**6/6:**	7.2 E-7	32.7	**6/6:**
Little Juniata River at mouth (MLJ)	A	1.6 E-7	36.2	0/6	6.1 E-8	36.5	0/6
Trough Creek (TC)	A	1.8 E-7	34.9	1/6:	9.4 E-8	35.9	0/6
Aughwick Creek (AC)	U	7.8 E-7	32.4	**6/6:**	3.7 E-8	36.6	0/6
Lower Juniata River (MJ)	P	3.0 E-6	30.2	**6/6:**	6.9 E-8	35.5	0/6

Avg. C_T,_ Average C_T_ values; C_T_ = 34, number of replicates that were positive by site with a C_T_ threshold of 34.

Bolded values represent sites deemed positive for *F*. *rusticus*.

^a^ A is putative absence, P is presence, and U is unknown.

In contrast, the combination of the DNeasy Blood and Tissue extraction kit and ethanol, only two of the three putative positive sites (upper Juniata River and Shaver’s Creek) indicated presence (Fig 1A, Table 2). Samples from the unknown and putative absent sites failed to generate amplification below the threshold and indicated absence for these areas.

Utilizing the Qiagen PowerWater extraction protocol and Longmire’s Buffer, samples from the three putative positive sites indicated presence (Fig 1B, Table 3). Samples from the sites of putative absence and the unknown site did not generate amplification below the C_T_ 34 threshold in > 3 replicates at any site. The combination of Qiagen PowerWater extraction and ethanol storage provided no positives (Fig 1B, Table 3). Amplification below the C_T_ threshold was not seen within any replicate for any sample, even in places of putative presence.

**Table 3:** Qiagen’s PowerWater extraction kit with a TaqMan qPCR assay.

PowerWater, TaqMan							
		Longmire’s Buffer			95% Ethanol		
Site	Presence^a^	Conc. (nmol)	Avg. C_T_	C_T_ = 34	Conc. (nmol)	Avg. C_T_	C_T_ = 34
Frankstown Branch Mouth (FM)	A	5.6 E-7	35.8	1/6			0/6
Upper Juniata River (UJ)	P	2.1 E-6	33.0	**5/6:**	1.8 E-7	36.2	0/6
Upper Little Juniata River (LJU)	A	2.8 E-7	37.0	1/6	1.4 E-7	37.7	0/6
Shaver’s Creek (SC)	B	2.2 E-5	29.3	**6/6:**	1.3 E-7	37.8	0/6
Little Juniata River at mouth (MLJ)	A	6.1 E-8	38.5	0/6	4.2 E-7	35.5	0/6
Trough Creek (TC)	A	5.0 E-7	35.6	1/6			0/6
Aughwick Creek (AC)	U	7.2 E-7	34.8	1/6	2.9 E-7	36.1	0/6
Lower Juniata River (MJ)	P	2.0 E-5	29.5	**6/6:**	7.9 E-8	38.6	0/6

Avg. C_T,_ Average C_T_ values; C_T_ = 34, number of replicates that were positive by site with a C_T_ threshold of 34.

Bolded values represent sites deemed positive for *F*. *rusticus*.

^a^ A is putative absence, P is presence, and U is unknown.

Table 3. Average concentration, C_T_ values, and number of replicates that were positive by site with a C_T_ threshold of 34 for filter samples extracted using Qiagen’s PowerWater kit with a TaqMan qPCR assay. Presence is noted as A for putative absence, P for presence, B for both *F*. *rusticus* and *F*. *obscurus*, and U for unknown. Bolded values represent sites deemed positive for *F*. *rusticus*.

Sites of putative presence did not all meet the threshold for the SYBR green assays. With the DNeasy Blood and Tissue extraction kit and Longmire’s storage buffer, only one of the three sites with putative presence (Shaver’s Creek) generated C_T_ values below the 32-cycle threshold (Fig 1C, Table 4). The site with unknown presence resulted in an average C_T_ below the 34-cycle threshold suggesting crayfish presence, and sites of putative absence did not result in > 3 replicates below this threshold. The combination of DNeasy Blood and Tissue extraction kit and ethanol generated similar results, with Shaver’s Creek and lower Juniata River being the only putative positive sites showing early amplification and the unknown and negative samples not amplifying below the C_T_ threshold (Fig 1C, Table 4).

**Table 4:** A Qiagen’s DNEasy extraction kit with a SYBR Green qPCR assay.

DNeasy, SYBR									
		Longmire’s Buffer				95% Ethanol			
Site	Presence^a^	Avg. T_M_	Conc. (nM)	Avg. C_T_	C_T_ = 32	Avg. T_M_	Conc. (nM)	Avg. C_T_	C_T_ = 32
Frankstown Branch Mouth (FM)	A	75.6	3.2 E-7	34.6	0/6	75.1	1.3 E-7	35.7	0/6
Upper Juniata River (UJ)	P				0/6	77.9	9.0 E-7	32.8	1/6
Upper Little Juniata River (LJU)	A	75.6	6.5 E-7	33.7	0/6	75.6	5.4 E-7	33.6	0/6
Shaver’s Creek (SC)	B	77.6	6.2 E-6	28.4	**6/6:**	77.7	3.5 E-6	29.2	**6/6**
Little Juniata River at mouth (MLJ)	A	76.1	9.4 E-8	32.9	0/6	77.4	5.9 E-7	33.0	1/6
Trough Creek (TC)	A	76.9	1.1 E-7	35.4	0/6	75.8	8.1 E-7	33.1	1/6
Aughwick Creek (AC)	U	76.7	1.7 E-6	30.8	**4/6**	76.4	1.3 E-6	33.1	1/6
Lower Juniata River (MJ)	P				0/6	77.2	6.0 E-7	32.4	**3/6**

T_M,_ average melting point; Avg. C_T,_ Average C_T_ values; C_T_ = 32, number of replicates that were positive by site with a C_T_ threshold of 32.

Bolded values represent sites deemed positive for *F*. *rusticus*.

^a^ A is putative absence, P is presence, and U is unknown.

The combination of Qiagen PowerWater extraction kit and Longmire’s buffer resulted in two of the three putative positive sites (Shaver’s Creek and lower Juniata River), along with samples from the presence-unknown site (Aughwick Creek) with C_T_ values below the 32-cycle cutoff (Fig 1D, Table 5). The putative negative sites did not result in any amplification below the C_T_ threshold apart from Frankstown Branch mouth which amplified below the C_T_ threshold in 3/6 replicates. Finally, the combination of Qiagen PowerWater extraction kit and ethanol storage yielded one positive site confirmation (upper Juniata River) with amplification lower than the threshold but included no other positive sites or the unknown site (Fig 1D, Table 5). One site of putative absence which resulted in a positive read for the previous SYBR PowerWater runs, Frankstown Branch Mouth, also generated an average C_T_ below the threshold for the majority of runs.

**Table 5:** Qiagen’s PowerWater extraction kit with a SYBR Green qPCR assay.

PowerWater, SYBR									
		Longmire’s Buffer				95% Ethanol			
Site	Presence^a^	Avg. T_M_	Conc. (nM)	Avg. C_T_	C_T_ = 32	Avg. T_M_	Conc. (nM)	Avg. C_T_	C_T_ = 32
Frankstown Branch Mouth (FM)	A	77.4	3.4 E-7	30.5	**6/6**	76.1	1.5 E-7	31.7	**4/6**
Upper Juniata River (UJ)	P	77.7	1.7 E-7	32.0	2/6	76.8	5.0 E-7	33.3	2/6:
Upper Little Juniata River (LJU)	A	81.3	3.7 E-8	35.3	0/6	74.4	8.0 E-8	32.4	1/6
Shaver’s Creek (SC)	B	80.1	6.0 E-7	28.2	**6/6:**	76.5	1.1 E-7	33.8	1/6
Little Juniata River at mouth (MLJ)	A	76.9	6.2 E-8	34.2	1/6	77.3	2.4 E-7	32.9	1/6:
Trough Creek (TC)	A	77.3	6.8 E-8	33.4	1/6	75.1	7.8 E-8	32.5	1/6
Aughwick Creek (AC)	U	77.4	9.2 E-7	29.1	**6/6:**	77.8	1.9 E-7	32.0	2/6:
Lower Juniata River (MJ)	P	77.5	2.5 E-6	27.3	**6/6:**	76.1	1.7 E-7	32.1	**3/6**

T_M,_ average melting point; Avg. C_T,_ Average C_T_ values; C_T_ = 32, number of replicates that were positive by site with a C_T_ threshold of 32.

Bolded values represent sites deemed positive for *F*. *rusticus*.

^a^ A is putative absence, P is presence, and U is unknown.

The melt-curves for the SYBR eDNA filter samples were highly varied and presented no notable trends. There was no clear distinction of T_M_ between samples collected in streams with putative presence of only *F*. *rusticus* as compared to samples collected at Shaver’s Creek, the site with both *F*. *rusticus* and *F*. *obscurus* present. The C_T_ values in the SYBR green eDNA assays showed high variation across replicates and less precision compared to the TaqMan assays and late amplification greater than the C_T_ cutoff threshold of 32 was likely a result of primer dimer.

## Discussion

Our eDNA assay using TaqMan primers and a probe for determining presence of *F*. *rusticus* showed sensitivity towards our target species when we tested against tissue samples of different local crayfish species. Further testing of storage buffer, DNA extraction kit, and qPCR chemistry found that the combination with the greatest agreement to putative information was filter storage in Longmire’s buffer, the DNeasy blood and tissue kit, and the TaqMan probe qPCR chemistry.

Tissue analyses with traditional PCR showed primer specificity to *F*. *rusticus*. A qPCR analysis using a TaqMan assay with DNA diluted from tissue samples confirmed that our primers were sensitive to *F*. *rusticus*, however, the SYBR assay amplified all three species of crayfish and did not show enough precision in the T_M_ to discriminate between species. Previously, Dougherty et al. [38] established a SYBR eDNA assay specific to *F*. *rusticus* in the upper Midwest, USA that resulted in non-existent amplification in 8 species and delayed amplification (up to 17.5 cycles) in the virile crayfish (*F*. *virilis)*. We used a slightly modified primer sequence due to the instability of the guanine tetrad included in their primer. The primer differences may account for the larger delay in non-target amplification in Dougherty et al. [38]. In addition to their use of a different primer, we suspect there may be slight differences in the COI sequence between the upper Midwest and Pennsylvania samples that explain this disparity.

In field testing, not all combinations of storage buffer and extraction techniques confirmed putative information. Only with the use of Longmire’s storage, DNeasy extraction, and TaqMan qPCR chemistry was all putative information confirmed. Although storage in ethanol has long been the norm, similar to others [33, 51] we found that Longmire’s buffer garnered more success in our assay. The combination of ethanol storage with PowerWater extraction seems to provide especially low quality results in analyses, yielding markedly lower DNA concentrations from qPCR [52] and no positive tests for our own studies when used in conjunction with TaqMan chemistry. Other aquatic eDNA studies have found that when using filtration methods, DNA extraction with DNeasy Blood and Tissue kit consistently garnered more reliable results than the PowerWater kit [35, 52, 53]. Goldberg et al. [29] asserts that in the comparison studies they analyzed, DNeasy worked better for extraction from cellulose nitrate filters and recommends use of probe-based assays, like TaqMan, for genotyped species as they allow for more sensitivity and specificity. The use of the TaqMan probe in our assay additionally allowed us to test inhibition with an IPC and garnered clearer results compared to SYBR analyses.

We point out that our eDNA assay most accurately represents known presence of *F*. *rusticus* when optimized storage and DNA extraction conditions are also used, and this highlights the importance of validation when developing or updating an eDNA assay. In the majority of our tests, regardless of technique, putative absent areas were confirmed while only certain combinations of buffer and DNA extraction techniques confirmed positive sites. Since three different combinations of qPCR chemistry, storage, and extraction techniques showed positive for the site of unknown presence, Aughwick Creek, we can confirm presence at this sampling location. Although Frankstown Branch mouth, a site of putative absence, resulted in positive reads with PowerWater extraction and SYBR qPCR chemistry, we believe this small subset of combinations showing presence is not enough to call the site positive as the SYBR and PowerWater combination does not reliably assign presence to sites with known *F*. *rusticus* occurrence. This also confirms what we have found from dip net sampling. From 2016 through 2019 we sampled the Frankstown Branch during 5 different sampling occasions for various purposes. We caught numerous crayfish, 34 of which were vouchered, and have not observed rusty crayfish.

This eDNA assay provides an update to Doughtery et al. [38] and shows that the TaqMan primer and probe chemistry is more reliable than the SYBR green assay in this system. This work also marks the first study showing success for *F*. *rusticus* eDNA detection in streams. Dougherty et al. [38] established an eDNA assay sensitive enough to detect the presence of *F*. *rusticus* eDNA in lakes in the upper Midwest, USA and were able to use this to detect *F*. *rusticus* in locations where it was previously not found. They found success, as did we, when sampling at or just below the water’s surface. Previous eDNA studies had difficulties with assays when sampling from pond sediments, as Tréguier et al. [54] had a low success rate when testing eDNA against areas of known presence for *Procambarus clarkii* in freshwater ponds. In our own sampling, we did not disturb the substrate, as the surface water has been shown to better represent current fish community composition in water systems while also likely having less inhibitory factors [26]. Reliable eDNA detection not only depends on where samples are taken but is also influenced by water levels and seasons. Sediments and sediment-associated bacteria can be resuspended during high stream-flow events, as shown by Jamieson et al. [55], and resuspension is also likely for benthic eDNA that has settled over time. This may interfere with time-sensitive detections, as eDNA does not linger in the surface water but can be maintained for up to 3 months in sediment [26]. When investigating behavioral and seasonal factors that may affect crayfish eDNA amounts, Dunn et al. [56] found that *Pacifastacus leniusculus* detection through eDNA was more successful when eggs were present. In looking for a relationship between eDNA and abundance in late summer and early fall, Dougherty et al. [38] found weak relationships between crayfish relative abundance and eDNA concentration at either the whole lake or the individual sample location scale.

The goal of this work was to establish an eDNA assay and protocol that can be used to accurately detect *F*. *rusticus* in central Pennsylvania. Using this eDNA-based assay allows for noninvasive monitoring that can be utilized to determine how far they have spread through the area. In addition to disrupting the structure of local ecosystems, we are especially concerned with the potential invasion through Raystown Lake and into the headwaters of the Raystown Branch, and into the Little Juniata River. The introduction of rusty crayfish here could be detrimental to the local recreational fishing economy, as *F*. *rusticus* often lead to the decline of the macroinvertebrates [10, 11, 57], which are key food items for trout [58, 59]. They are also known to consume and significantly reduce fish eggs and could negatively impact trout recruitment [60]. Our eDNA assay is a good first step towards mapping the current invasion front and developing management strategies to control *F*. *rusticus* in the Juniata River watershed.

## Supporting information

S1 Fig Calibration curve plot for the TaqMan qPCR assay depicting the Limit of Detection (LOD) and Limit of Quantification (LOQ). (DOCX)

S2 Fig Limit of Detection (LOD) plot for TaqMan qPCR assay with detection probability and 95% CI for replicate analyses. (DOCX)

S3 Fig Limit of Quantification (LOQ) plot for the TaqMan qPCR assay with blue line representing the LOQ model, red line representing Limit of Detection (LOD) and the calculated LOQ is the gray rectangle where it hits the model curve with the precision threshold (0.35 CV) defined by the upper limit of the rectangle. (DOCX)

S4 Fig Calibration curve plot for the SYBR qPCR assay depicting the Limit of Detection (LOD) and Limit of Quantification (LOQ). (DOCX)

S1 Table Amplification results from in silico testing with Sequence Manipulation Suite (Stothard 2000) using the primer FaRu and GenBank COI results. (DOCX)
