# Supplementary material for: The update and optimization of an eDNA assay to detect the invasive rusty crayfish (Faxonius rusticus)
Source: PLoS One. 2021 Oct 29;16(10):e0259084. doi: 10.1371/journal.pone.0259084 (PMC8555798; doi:10.1371/journal.pone.0259084)
Supplement: S2 Fig — (DOCX) [file pone.0259084.s002.docx]

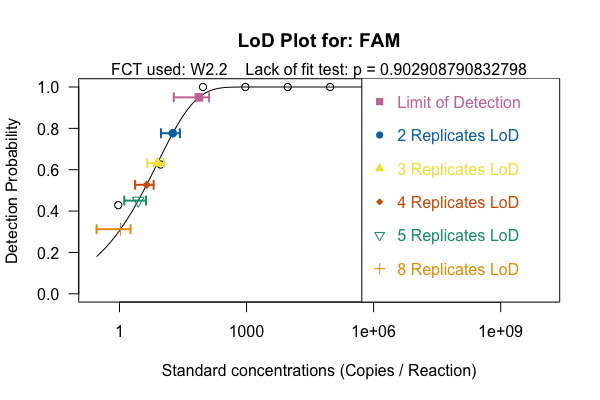


**S2 Fig. Limit of Detection (LOD) plot for TaqMan qPCR assay with detection probability and 95% CI for replicate analyses.**
