# Supplementary material for: The update and optimization of an eDNA assay to detect the invasive rusty crayfish (Faxonius rusticus)
Source: PLoS One. 2021 Oct 29;16(10):e0259084. doi: 10.1371/journal.pone.0259084 (PMC8555798; doi:10.1371/journal.pone.0259084)
Supplement: S3 Fig — (DOCX) [file pone.0259084.s003.docx]

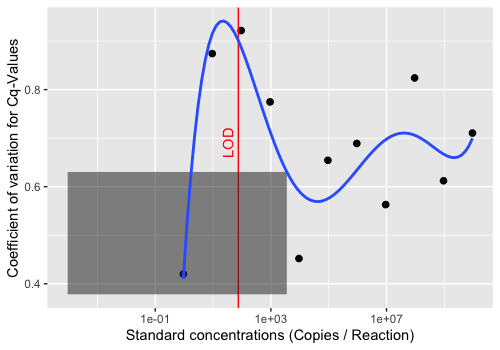


**S3 Fig. Limit of Quantification (LOQ) plot for the TaqMan qPCR assay with blue line representing the LOQ model, red line representing Limit of Detection (LOD) and the calculated LOQ is the gray rectangle where it hits the model curve with the precision threshold (0.35 CV) defined by the upper limit of the rectangle.**
