# Supplementary material for: The update and optimization of an eDNA assay to detect the invasive rusty crayfish (Faxonius rusticus)
Source: PLoS One. 2021 Oct 29;16(10):e0259084. doi: 10.1371/journal.pone.0259084 (PMC8555798; doi:10.1371/journal.pone.0259084)
Supplement: S1 Table — (DOCX) [file pone.0259084.s005.docx]

**S1 Table. Amplification results from in silico testing with Sequence Manipulation Suite (Stothard 2000) using the primer FaRu and GenBank COI results.**

| Species | GenBank Acc. # | Amplified | Amplicon |
| --- | --- | --- | --- |
| Faxonius rusticus | AY701249 | Y | GGGCGTCAGTAGATTTAGGTATTTTTTCGTTACATTT  AGCCGGGGTTTCTTCTATTCTTGGCTCAGTTAATTTTA  TAACAACGGCTATTAATATACGAGCTACGGGAATGAC |
| Faxonius obscurus | KT759576.1 | N |  |
| *Faxonius immunis* | DQ882095.1 | N |  |
| Faxonius virilis | MK903538.1 | N |  |
| *Faxonius propinquus* | KM611648.1 | N |  |
| *Faxonius limosus* | MH087572.1 | N |  |
| *Cambarus bartonii* | KT759646.1 | N |  |
| *Cambarus speciosus* | JX514459.1 | N |  |
| *Cambarus diogenes* | MH087704.1 | N |  |
| *Cambarus acuminatus* | MK410968.1 | N |  |
| *Cambarus carinirostris* | JX514436.1 | N |  |
| *Cambarus robustus* | KY712448.1 | N |  |
| *Procambarus acutus* | MK308298.1 | N |  |
| *Procambarus clarkii* | KT959364.1 | N |  |
